# Association of Essential Tremor With Novel Risk Loci: A Genome-Wide Association Study and Meta-analysis

**DOI:** 10.1001/jamaneurol.2021.4781

**Published:** 2022-01-04

**Authors:** Calwing Liao, Charles-Etienne Castonguay, Karl Heilbron, Veikko Vuokila, Miranda Medeiros, Gabrielle Houle, Fulya Akçimen, Jay P. Ross, Helene Catoire, Monica Diez-Fairen, Jooeun Kang, Stefanie H. Mueller, Simon L. Girard, Franziska Hopfner, Delia Lorenz, Lorraine N. Clark, Alexandra I. Soto-Beasley, Stephan Klebe, Mark Hallett, Zbigniew K. Wszolek, Manuela Pendziwiat, Oswaldo Lorenzo-Betancor, Klaus Seppi, Daniela Berg, Carles Vilariño-Güell, Ronald B. Postuma, Geneviève Bernard, Nicolas Dupré, Joseph Jankovic, Claudia M. Testa, Owen A. Ross, Thomas Arzberger, Sylvain Chouinard, Elan D. Louis, Paola Mandich, Carmine Vitale, Paolo Barone, Elena García-Martín, Hortensia Alonso-Navarro, José A. G. Agúndez, Félix Javier Jiménez-Jiménez, Pau Pastor, Alex Rajput, Günther Deuschl, Gregor Kuhlenbaümer, Inge A. Meijer, Patrick A. Dion, Guy A. Rouleau

**Affiliations:** 1 Department of Human Genetics, McGill University, Montreal, Quebec, Canada; 2 Montreal Neurological Institute, McGill University, Montreal, Quebec, Canada; 3 Department of Neurology and Neurosurgery, McGill University, Montreal, Quebec, Canada; 4 23andMe, Inc, Sunnyvale, California; 5 Fundació Docència i Recerca Mútua Terrassa, University Hospital Mútua de Terrassa, Terrassa, Barcelona, Spain; 6 Movement Disorders Unit, Department of Neurology, University Hospital Mútua de Terrassa, Terrassa, Barcelona, Spain; 7 Division of Genetic Medicine, Department of Medicine, Vanderbilt Genetics Institute, Vanderbilt University Medical Center, Nashville, Tennessee; 8 Institute of Health Informatics, University College London, London, United Kingdom; 9 Département des Sciences Fondamentales, Université du Québec à Chicoutimi, Saguenay, Quebec, Canada; 10 Montreal Neurological Institute, Department of Neurology and Neurosurgery, McGill University, Montreal, Quebec, Canada; 11 Department of Neurology, Hannover Medical School, Hannover, Germany; 12 University Children’s Hospital, University of Würzburg, Wurzburg, Germany; 13 Department of Pathology and Cell Biology, Taub Institute, Columbia University, New York, New York; 14 Department of Neurology, Mayo Clinic Florida, Jacksonville; 15 Department of Neurology, University Hospital Würzburg, Wurzburg, Germany; 16 Department of Neurology, University Hospital Essen, Essen, Germany; 17 National Institute of Neurological Disorders and Stroke Intramural Research Program, National Institutes of Health, Bethesda, Maryland; 18 Department of Neurology, Mayo Clinic Florida, Jacksonville; 19 Institute of Clinical Molecular Biology, University of Kiel, Kiel, Germany; 20 Department of Neuropediatrics, University Medical Center Schleswig-Holstein, University of Kiel, Kiel, Germany; 21 Veterans Affairs Puget Sound Health Care System, Seattle, Washington; 22 Department of Neurology, University of Washington School of Medicine, Seattle; 23 Department of Neurology, Innsbruck Medical University, Innsbruck, Austria; 24 Department of Neurology, University Hospital Schleswig-Holstein, University of Kiel, Kiel, Germany; 25 Department of Medical Genetics, University of British Columbia, Vancouver, British Columbia, Canada; 26 Division of Pediatric Neurology, Departments of Pediatrics, Neurology and Neurosurgery, Montreal Children’s Hospital, Montreal, Quebec, Canada; 27 Child Health and Human Development Program, Research Institute of the McGill University Health Centre, Montreal, Quebec, Canada; 28 Division of Medical Genetics, Department of Specialized Medicine, Montreal Children’s Hospital, McGill University Health Centre, Montreal, Quebec, Canada; 29 Faculté de Médecine, Université Laval, Centre Hospitalier Universitaire de Québec (l’Enfant-Jésus), Quebec, Canada; 30 Parkinson’s Disease Center and Movement Disorders Clinic, Department of Neurology, Baylor College of Medicine, Houston, Texas; 31 Parkinson’s and Movement Disorders Center, Department of Neurology, Virginia Commonwealth University, Richmond; 32 Departments of Neuroscience and Clinical Genomics, Mayo Clinic Florida, Jacksonville; 33 Department of Psychiatry and Psychotherapy, University Hospital, Ludwig-Maximilians-University Munich, Munich, Germany; 34 Center for Neuropathology and Prion Research, Ludwig-Maximilians-University Munich, Munich, Germany; 35 Unité des troubles du mouvement André Barbeau, Centre Hospitalier de l’Université de Montréal, Montreal, Quebec, Canada; 36 Department of Neurology, The University of Texas Southwestern Medical Center, Dallas; 37 Department of Neuroscience, Rehabilitation, Ophthalmology, Genetics and Maternal and Child Health (DINOGMI), University of Genoa, Genova, Italy; 38 Istituto di Ricovero e Cura a Carattere Scientifico Policlinico, San Martino, Genova, Italy; 39 Department of Motor Sciences and Wellness, University Parthenope, Naples, Italy; 40 Center for Neurodegenerative Disease (CEMAND), Department of Medicine, Surgery and Dentistry, Scuola Medica Salernitana, University of Salerno, Baronissi, Salerno, Italy; 41 University Institute of Molecular Pathology Biomarkers, UNEx, ARADyAL Instituto de Salud Carlos III, Caceres, Spain; 42 Section of Neurology, Hospital Universitario del Sureste, Arganda del Rey, Madrid, Spain; 43 University of Saskatchewan, Saskatoon Health Authority, Saskatoon, Saskatchewan, Canada; 44 Department of Neurology, University Medical Center Schleswig Holstein, University of Kiel, Kiel, Germany; 45 Department of Neuroscience and Pediatrics, Université de Montréal, Montreal, Quebec, Canada

## Abstract

**Importance:**

Essential tremor (ET) is one of the most common movement disorders, affecting 5% of the general population older than 65 years. Common variants are thought to contribute toward susceptibility to ET, but no variants have been robustly identified.

**Objective:**

To identify common genetic factors associated with risk of ET.

**Design, Setting, and Participants:**

Case-control genome-wide association study. Inverse-variance meta-analysis was used to combine cohorts. Multicenter samples collected from European populations were collected from January 2010 to September 2019 as part of an ongoing study. Included patients were clinically diagnosed with or reported having ET. Control individuals were not diagnosed with or reported to have ET. Of 485 250 individuals, data for 483 054 passed data quality control and were used.

**Main Outcomes and Measures:**

Genotypes of common variants associated with risk of ET.

**Results:**

Of the 483 054 individuals included, there were 7177 with ET (3693 [51.46%] female; mean [SD] age, 62.66 [15.12] years), and 475 877 control individuals (253 785 [53.33%] female; mean [SD] age, 56.40 [17.6] years). Five independent genome-wide significant loci and were identified and were associated with approximately 18% of ET heritability. Functional analyses found significant enrichment in the cerebellar hemisphere, cerebellum, and axonogenesis pathways. Genetic correlation (*r*), which measures the degree of genetic overlap, revealed significant common variant overlap with Parkinson disease (*r*, 0.28; *P* = 2.38 × 10^−8^) and depression (*r*, 0.12; *P* = 9.78 × 10^−4^). A separate fine-mapping of transcriptome-wide association hits identified genes such as *BACE2*, *LRRN2*, *DHRS13*, and *LINC00323* in disease-relevant brain regions, such as the cerebellum.

**Conclusions and Relevance:**

The results of this genome-wide association study suggest that a portion of ET heritability can be explained by common genetic variation and can help identify new common genetic risk factors for ET.

## Key Points

**Question:**

Can common genetic variants associated with essential tremor (ET) be identified?

**Findings:**

In this genome-wide association study and meta-analysis including genetic data on 483 054 individuals, 5 genome-wide significant loci were associated with risk of ET and common variants were associated with approximately 18% of ET heritability.

**Meaning:**

Findings of this study may help identify new genes and inform ET biology.

## Teaser

This genome-wide association study identifies common variants associated with risk of essential tremor.

## Introduction

Essential tremor (ET) is a complex neurological disorder affecting 1% of the general population and up to 5% of individuals older than 65 years.^1,2^ ET is clinically characterized as a bilateral, largely symmetric kinetic or postural tremor,^3^ which can greatly decrease the quality of life and debilitate daily function. Previous studies have implicated the cerebellum as a putative region of interest for ET.^4,5^ Specifically, abnormalities of Purkinje cells have been observed in postmortem brain tissue obtained from individuals with ET.^6^ Several transcriptomic studies and imaging studies have also highlighted the importance of the cerebellum in ET.^4,7,8^

The genetic etiology of ET remains elusive, despite twin studies that have shown the trait to be heritable.^9,10,11^ For instance, 1 twin study indicated ET had a concordance of 69% to 93% in monozygotic twins and 27% to 29% in dizygotic twins.^9^ Studies in which individuals with ET in the same family were sequenced have also implicated specific genes.^12,13,14^ For instance, rare variants in *FUS* and *TENM4* were found to segregate in large families but the lack of replication suggests they are potentially private variants.^15,16^ Past genome-wide association studies (GWAS) have also identified putative ET loci, but none of these loci were statistically significant at a genome-wide level, likely owing to the size of the cohorts examined.^17,18^ The loci from these GWAS implicated nearby genes, such as *STK32B* and *LINGO1*, for which subsequent replication studies were conducted.^19,20,21,22,23^ However, most of these GWAS loci had conflicting replication results and were conducted in smaller cohorts.

Here, we present a genome-wide meta-analysis identifying the first genome-wide significant loci for ET using a cohort of 7177 individuals with ET and 475 877 control individuals. We additionally identified novel loci, implicated tissue-relevant genes, and found a significant genetic overlap between Parkinson disease (PD) and ET. This report supported the heritable nature of ET and implicated new disease-relevant loci and genes.

## Methods

### Statistical Analyses

#### Sample Description

The meta-analysis included cohorts in North America and Europe, totaling 7177 individuals with ET and 475 877 control individuals. This population was further grouped into cohorts based on study cohort, chip, and time of genotyping as described in the eMethods in Supplement 1. Individual clinical diagnoses are described previously or in the eMethods in Supplement 1. The review board at the McGill University Health Center Research Ethics Board approved the study protocols (reference number: IRB00010120). Written informed consent was obtained from all participants.

#### Genotyping and Quality Control

The cohorts were genotyped and followed standardized quality control, imputation, and postimputation quality control. Samples were removed if there was greater than 2% missingness, autosomal heterozygous deviation (*F* < 0.2), or failed sex check. Low quality single-nucleotide variants (SNVs) were removed based on Hardy-Weinberg equilibrium (*P* > 1 × 10^−6^) and SNV missingness less than 0.02 after sample removal. Samples were mapped against the 1000 Genomes Project phase 3 reference panel after pruning and removing SNVs from high–linkage disequilibrium (LD) regions, and only individuals of inferred European ancestry were retained owing to low sample size for individuals of non-European ancestry. No relatedness filter was applied because a linear mixed model was subsequently used to account for relatedness. Imputation was done using the Sanger Imputation Server with Eagle version 2.3.5 and the Haplotype Reference Consortium Reference Panel version 1.1.^24^ Further details on cohort and quality control for the UK BioBank and 23andMe data sets are described in the eMethods in Supplement 1.

#### Genome-Wide Association

A bayesian linear mixed model was done using BOLT-LMM 2.3.4, including 20 principal components and sex as covariates to accelerate convergence.^25^ The noninfinitesimal model was used if there was an increase in power. Subsequently, the data were meta-analyzed using an inverse-variance–weighted fixed-effects model with METAL.^26^ Only markers with an effective sample size N = 4 / (1 / No. affected + 1/No. controls) > 70% were retained, leaving a total of 6 892 661 variants (eTable 12 in Supplement 9).^27^

#### SNV Heritability and Partitioning of Heritability

To determine SNV heritability on the liability scale, the slope of the LD Score regression (LDSC) was calculated with individuals of European ancestry from the 1000 Genomes Project.^28^ The effects of confounding factors were determined by assessing the deviation of the LDSC intercept from 1. Specifically, the ratio between the (intercept −1) divided by the (mean χ^2^ − 1) indicated confounding other than polygenicity.^28^ Heritability was partitioned by different tissue, cell, and functional sets using the LDSC.^29^ The 1000 Genomes Project cohort was used for LD and allele frequencies.

#### Genetic Correlation

Genetic correlation was calculated for ET and other GWAS traits using LD Hub.^30^ This platform uses the LDSC to broadly assess multiple traits with publicly available GWAS. Traits with an updated GWAS were replaced, as defined by a larger sample size and/or a more recently published GWAS. Only traits with European ancestry were retained and data with low relative *z* scores (as reported by LD Hub) were excluded. SNVs from the major histocompatibility complex region were removed for traits. One of any duplicate traits was retained, prioritizing the most recent study or largest sample size.

#### Conditional Analysis

To determine whether there were any genome-wide significant loci with multiple independent signals, genome-wide complex trait analysis–conditional and joint analysis was used.^31^ The program takes the ET summary statistics and conditions genome-wide significant lead SNVs while using the LD of a reference panel. Here, the raw genotyping data from control samples and the CARTaGENE cohort were used as the reference panel.^32^ A stepwise approach was used to condition the top independent SNVs (*P* < 5 × 10^−8^) and a minor allele frequency greater than 0.01.

#### Multitrait Analysis

To increase power, we did a multitrait analysis of GWAS (MTAG), using phenotypes with significant positive correlation (PD and depression).^33,34,35^ The MTAG program was used to conduct the analysis. MTAG jointly meta-analyzed summary statistics from PD and depression with ET to increase power to identify ET-specific associations. Increase in power was defined as (multitrait analysis GWAS mean χ^2^ −1) / (non–multitrait analysis GWAS mean χ^2^ −1) × 100%.

#### Multitrait Conditional Analysis

To assess the association between PD and ET, a multitrait conditional analysis was done using multitrait conditional and joint analysis, adjusting ET by PD. Summary statistics from Nalls et al^34^ were used. Multitrait conditional and joint analysis removed pleiotropic signal with PD from the ET GWAS. Typically, most pleiotropic loci should have reduced conditional effect sizes, but trait-specific effects would have larger conditional effects.

#### Gene-Based, Gene-Set, and Tissue-Set Enrichment Analyses

*P* values that quantify the genic associations and gene-set enrichment for ET were calculated using MAGMA version 1.08 as implemented in FUMA (https://fuma.ctglab.nl).^36,37^ Bonferroni correction was applied for the number of genes (N = 18 517) tested with a threshold of *P* = 2.70 × 10^−6^. Enrichment among GTEx version 8 was also done using FUMA, with a significance threshold of *P* = 9.26 × 10^−4^.

#### Transcriptome-Wide Association

To identify genes influenced by cis-eQTLs, a transcriptome-wide association study (TWAS) was done using FUSION. Brain imputation panels were used from the Genotype-Tissue Expression (GTEx) project and the CommonMind Consortium.^38,39^ The 1000 Genomes Project version 3 LD panel was used for TWAS. Bonferroni-adjusted *P* values less than .05 were considered transcriptome-wide significant. A brain omnibus test was done to test for effect across reference panels, which accounts for pairwise correlation between features. A Bonferroni threshold was used for the omnibus (0.05 / 7221) × (number of genes tested).

To address coregulation in TWAS, fine-mapping of causal gene sets (FOCUS) was used for genome-wide significant loci to model predicted expression correlations and assign a posterior probability for causality in the previously mentioned imputation panels.^40^ FOCUS identified genes for each TWAS signal to be included in a 90% credible set.

Gene-set analyses were done using GeneNetwork version 2.0 (https://genenetwork.nl), which leverages RNA-sequencing data (n = 31 499) to provide coregulated genes within each pathway.^41^ Genes meeting a Bonferroni-adjusted *P* value less than .05 were used. Agnostic analyses of pathways in databases such as Reactome and GO were used.

To assess potential colocalization between the top significant loci with eQTLs, FUMA was used to map eQTLs with the top significant loci. Data from GTEx 53 version 8 for brain tissue and the CommonMind Consortium were used. All SNV-gene pairs of cis-eQTLs that were nominally significant were included (*P* < .05). The sign of the original eQTL data indicates the direction of effect for the tested allele. The lead SNVs from the 5 genome-wide significant loci were compared with the eQTL data.

#### Phenome-Wide Association

To investigate whether any top loci were associated with other phenotypes, the SNVs were assessed on the genetics Open Targets Platform (http://genetics.opentargets.org) and a PheWeb for the UK BioBank imaging data (https://open.win.ox.ac.uk/ukbiobank/big40/pheweb/).

#### Bivariate Gaussian Mixture Modeling of Polygenicity

To determine the univariate estimate of non-null SNVs (polygenicity) and shared polygenicity with PD, MiXeR version 1.3 was used on the summary statistics of ET and PD.^42^ In the cross-trait analysis, MiXeR modeled additive genetic effects as a combination of the following components: SNVs not influencing either ET or PD, SNVs influencing only 1 of 2 traits, or SNVs influencing both traits. After fitting the model, the dice coefficient (a parameter that estimates the proportion of overlapping variants), was calculated.

## Results

### Genome-Wide Significant ET Risk Loci

For the GWAS, samples from 14 different clinical centers and 2 biobanks were included (eMethods in Supplement 1). The cohorts were divided by genotyping array, leading to a total of 4 genotyping cohorts. The first clinical cohort was genotyped on the Axiom Genome-Wide CEU 1 Array (Affymetrix). The second clinical cohort was genotyped on the Illumina GSA array. The 2 biobank cohorts were from 23andMe and the UK BioBank. Prior to analysis, stringent quality control was performed on the data and ancestrally predicted Europeans were retained based on the 1000 Genomes Project phase 3 reference panel. Independent cohorts were meta-analyzed using an inverse-variance–weighted fixed-effects model. Of the 483 054 samples included, there were 7177 individuals with ET (3693 [51.46%] female; mean [SD] age, 62.66 [15.12] years), and 475 877 control individuals (253 785 [53.33%] female; mean [SD] age, 56.40 [17.6] years). Subsequently, variants with an effective sample size greater than 70% of the full meta-analysis were retained, leaving 6 892 661 markers.

From the meta-analyzed GWAS, the heritability explained by SNVs, was estimated to be 0.1829 (standard error [SE], 0.0141) using LDSC. The LDSC intercept, which indicates the degree of inflation due to confounding, was 1.051 (SE, 0.00074), which suggests low levels of confounding. The attenuation ratio, which assesses the degree of inflation due to polygenicity instead of confounding, was 0.14 (SE, 0.036), suggesting most inflation was due to polygenicity (eFigure 1 in Supplement 1).^28^ The genomic inflation factor (λ_1000_) was also determined to be 1.01. Genetic correlation, which captures the degree of genetic overlap with another cohort or trait, was calculated between cohorts using LDSC. Across the cohorts, correlations were significant and positive, providing evidence that effects were consistent across cohort designs (eTable 1 in Supplement 1). The clinical cohorts had a genetic correlation of 0.88 (SE, 0.20; *P* = 1.86 × 10^−5^), and these clinical cohorts respectively had a genetic correlation of 0.96 ± 0.148 (*P* = 9.34 × 10^−11^) and 0.52 ± 0.14 (*P* = 1.21 × 10^−4^) with the 23andMe cohort (eTable 1 in Supplement 1). The lower genetic correlation of 0.52 was between the clinical samples genotyped on the Illumina Array and 23andMe. Owing to the small effective sample size of the UK Biobank cohort, pairwise genetic correlation was not calculated.

**Figure 1.**
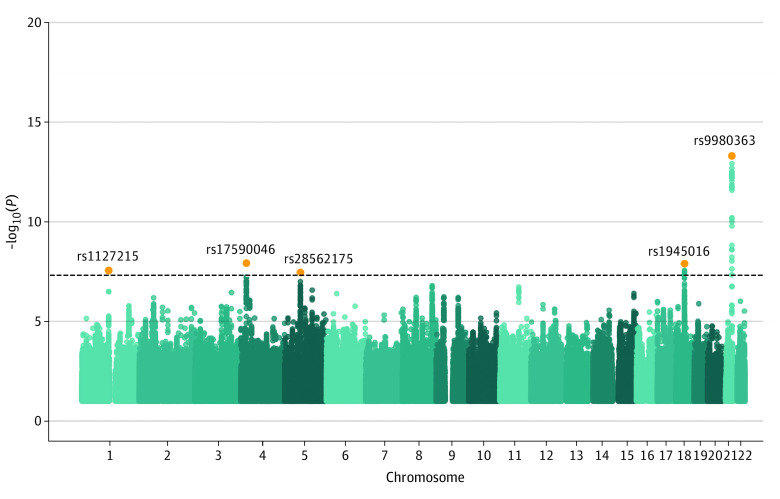
Manhattan Plot of Genome-Wide Association Study of 7177 Individuals With Essential Tremor and 475 877 Control Individuals The x-axis shows chromosome number (chromosomes 1-22) and points are ordered by genomic position. The y-axis shows the statistical significance of each variant, represented as -log10(P). The black-dashed line indicates the genome-wide significance threshold *P *= 5 × 10-8). Lead SNPs are highlighted in orange.

A total of 5 genome-wide significant loci (*P* < 5 × 10^−8^) were identified (Figure 1; Table). None of the of the top loci were found to be significantly heterogeneous (eTable 2 in Supplement 1). Furthermore, there were no additional independent secondary genome-wide significant signals found after conditioning on the lead SNVs iteratively.

**Table.** Lead Single-Nucleotide Variants (SNVs) for Genome-Wide Significant Loci in 7177 Individuals With Essential Tremor and 475 877 Control Individuals

CHR	Position (hg19)	rsID	Alleles (eff/ref)	Odds ratio (95% CI)	*z *Score	*P* value
1	117532790	rs1127215	C/T	1.09 (1.06 to 1.13)	5.55	2.756 × 10^−08^
4	24362541	rs17590046	C/T	0.89 (0.85 to 0.93)	−5.70	1.180 × 10^−08^
5	67827456	rs28562175	C/T	0.92 (0.88 to 0.95)	−5.51	3.483 × 10^−08^
18	37207175	rs1945016	G/T	1.10 (1.06 to 1.13)	5.69	1.255 × 10^−08^
21	42520134	rs9980363	C/T	1.16 (1.12 to 1.20)	7.52	4.921 × 10^−14^

Abbreviations: CHR, chromosome; eff, effective allele; hg19, human genome version 19; ref, reference; rsID, reference SNV number.

### Transcriptome-Wide Associations

**Figure 2.**
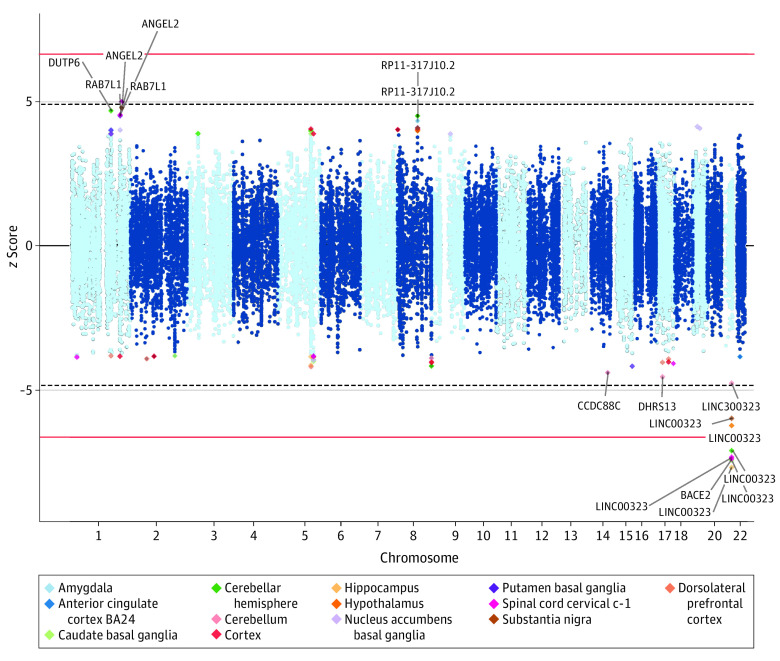
Mirrored Manhattan Plot of Transcriptome-Wide Associations for Essential Tremor The red line indicates Bonferroni-significance threshold. The dashed blue line indicates the false-discovery rate threshold.

A TWAS was conducted using FUSION by leveraging brain data from the GTEx and the CommonMind Consortium.^38,39,43^ The TWAS identified genes that were predicted to have altered their expression due to ET-associated common variants. The Bonferroni-significant hits were *BACE2* and *LINC00323* (Figure 2; eTable 3 in Supplement 2). A brain omnibus test, which assesses the degree of shared signal across brain tissues, showed that *BACE2*, *LINC00323*, and *ANGEL2* were significant after Bonferroni correction, suggesting effect across multiple brain tissue types (eTable 4 in Supplement 3).

To prioritize the most genes, TWAS fine-mapping was done using FOCUS across the set of 90%-credible genes.^40^ FOCUS models the correlation among TWAS signals so that the likely causal gene(s) at genome-wide significant loci are prioritized. Across the genome-wide significant loci, *BACE2* was prioritized with a posterior inclusion probability of 0.80 (*z* = −7.09) (eTable 5 in Supplement 4).

Additionally, the top significant loci were mapped to eQTLs derived from the GTEx 53 brain tissues and the CommonMind Consortium. The colocalization pointed toward three genes, *PTGFRN*, *LINC00323*, and *BACE2*. The *PTGFRN* gene was only significant in the cerebellum, whereas the latter 2 genes were implicated across multiple brain tissues (eTable 6 in Supplement 5).

### Functional Enrichment of Genomic Regions

To identify patterns of heritability from the GWAS data, heritability was partitioned by function annotations using partitioned LD score regression.^29^ This analysis indicated that there were significant enrichments from SNVs in H3K9ac peaks, H3K27ac, and conserved regions (eTable 7 in Supplement 1).

Moreover, genome-wide analyses were done using MAGMA, which aggregates significance across loci into gene-level significance.^36^ The top gene set identified after enrichment was axonogenesis (Bonferroni *P* = 0.047) (eTable 8; eFigures 2 and 3 in Supplement 1). There was significant enrichment in the cerebellum (*P* = 6.3 × 10^−5^) and cerebellar hemisphere (*P* = 7.9 × 10^−5^) in GTEx 53 and overall enrichment in the brain (*P* = .001) (eFigure 4 in Supplement 1).^37^ There was no significant gene enrichment across the 29 different ages in the BrainSpan cohort (eFigure 5 in Supplement 1).^44^

### Phenome-Wide Associations of Top Loci

For the genome-wide significant loci, a phenome-wide association study (pheWAS) was done to identify putatively relevant phenotypes. For the top *BACE2* locus associated with ET (rs9980363), the pheWAS of brain imaging data showed it was a top significant association for white matter intracellular volume fraction in the left and right inferior cerebellar peduncle (β, 0.08; SE, 0.012; *P* = 1.2 × 10^−12^).

### Genetic Correlation

To determine whether ET had a significant genome-wide genetic correlation with other diseases and traits, LD Hub and LDSC were used.^28,30^ After correcting for multiple testing, ET was shown to have a significant genetic correlation with PD (ρ_g_, 0.28; SE, 0.051; *P* = 6.44 × 10^−8^) and depression (ρ_g_, 0.12; SE, 0.04; *P* = 9.78 × 10^−4^) (eTable 9 in Supplement 6).

### Association Between ET and PD

Considering the epidemiological implications and positive genetic correlation that have been reported between ET and PD, we sought to further dissect their genetic association. MiXer, a bivariate causal mixture model, was used to estimate the number of causally shared SNVs. It was found that there were 500 causally shared SNVs between PD and ET, with a total of 700 variants that influence ET and 4800 that influence PD.

To determine whether the genome-wide significant signals were pleiotropic for ET and PD, multitrait conditional and joint analysis using GWAS summary data was used to estimate the SNV effect size of the outcome trait (ET) after conditioning on exposure trait (PD).^31^ To do this, multitrait conditional and joint analysis takes the PD genome-wide significant loci to estimate the effect of exposure on ET, then undergoes a genome-wide conditioning with the estimated effect for the outcome trait.^31^ All the genome-wide significant ET loci remained significant after conditioning, suggesting no pleiotropy with PD for these loci (eTable 10 in Supplement 7).

To assess whether novel ET loci could be identified given the genetic correlation with PD, a multitrait association analysis was done using MTAG (multitrait analysis of genome-wide association studies).^33^ This method leverages the genetic correlation between traits to increase power for each respective phenotype. An additional genome-wide significant locus was identified through MTAG on chromosome 3 (lead SNV: rs703174), with an increase in power up to 8.5% (eTable 11 in Supplement 8).

## Discussion

This genome-wide association study identified 5 genome-wide significant loci for ET, demonstrating the importance of common variants. One of the signals on chromosome 4 had nominal significance in a previous GWAS and was found to be consistent across the other cohorts included in this meta-analysis.^17^ The previous largest GWAS did not find any genome-wide significant loci that met the multiple-testing significance threshold but found 3 suggestive loci.^24^

For the chromosome 1 locus (rs1127215), the UK Biobank cohort did not have a consistent direction with the other cohorts. This may be because of bias from population biobanks, batch effects, and smaller case count. Interestingly, the UK Biobank cohort also had a low prevalence (approximately 0.06%), despite the expected prevalence of 1% to 5%. This may suggest an underreporting of ET in biobank surveys or a bias where there was decreased participation of patients with ET in the UK Biobank. Our study revealed multiple characteristics about the genetic architecture of ET. SNV-based heritability was found to be 18.29% on the liability scale, suggesting that a considerably large portion of heritability is explained by common variants. This is comparable with a variety of other brain-relevant disorders such as bipolar disorder, intracranial aneurysms, and PD.^34,45,46^ We also found this heritability to be enriched in histone markers such as H3K9ac and H3K27ac, which suggest future studies could investigate the importance of epigenetics for ET. We found an additional novel locus by leveraging the genetic overlap of PD with MTAG, which may suggest this locus is pleiotropic for the 2 phenotypes. Through gene-set enrichment analysis, we identified axonogenesis as an important cellular process for the disease, consistent with previous studies that implicate the importance of axons.^20,47,48,49^ Furthermore, we found significant associations between ET and the cerebellum, providing further evidence that ET may be a cerebellar disorder or reflective of neurons driving the signal due to high proportion of neurons in the cerebellum.^50,51,52,53^

A transcriptome-wide association study using brain cis-eQTL data from GTEx and the CommonMind Consortium found *BACE2*, *LINC00323*, and *ANGEL2* to be transcriptome-wide significant for ET. Probabilistic fine mapping further prioritized *BACE2* among the TWAS signals. The eQTL mapping also found converging evidence and implicated *BACE2*. The gene *BACE2* encodes for a β-secretase homologue that is capable of cleaving amyloid β precursor protein resulting in the formation of amyloid-β protein, a major component in the pathogenesis of Alzheimer disease.^54,55,56^ Interestingly, a postmortem study has found increased levels of insoluble and soluble amyloid-β protein in the cerebellar and parietal cortex of patients with ET compared with control individuals and patients with PD.^57^

Moreover, ET was found to be significantly genetically correlated with depression and PD, suggesting common variant overlap. Previous studies have found that ET is associated with both self-reported depression and antidepressant medication use, concordant with the genetic correlation results.^58^ We conditioned ET by PD and found an attenuation of genome-wide association strength but that the top ET loci were still genome-wide significant. This result suggests that these loci are likely to be robustly associated with ET and not with PD.

Phenome-wide association analysis of these loci revealed that the *BACE2* locus was associated with increased intracellular volume fraction, a marker of neuronal density, in the inferior cerebellar peduncles. The inferior cerebellar peduncles harbors the main afferent fibers of the cerebellum, channeling proprioceptive information from the spinal cord and brain stem nuclei to the cerebellar cortex.^59^ A recent volumetric analysis of magnetic resonance imaging brain scans found that the middle and inferior peduncles of the cerebellum of patients with ET displayed significant atrophy compared with healthy control individuals.^60^ In addition, stimulation of these afferent proprioceptive fibers was shown to be effective at reducing tremors in patients with ET.^61^ Interestingly, another study showed increased radial diffusivity, a parameter strongly associated with myelin abnormalities, in the inferior cerebellar peduncles of patients with ET.^62^ These results, paired with our findings of enrichment of genes in this region of the cerebellum and axonogenesis, highlight the potential implication of inferior cerebellar peduncles afferent fibers in ET pathophysiology.

### Limitations

This study has limitations, including the lack of diverse ancestry. We did not have a large number of non-European samples, which prevented us from conducting multiancestral analyses. Moreover, there is a lack of deep phenotyping information for population-based biobanks such as 23andMe, which may lead to an increased number of diagnostic inaccuracies at the expense of increased power. However, there was still a correlation with the clinical cohorts, suggesting that the population-based biobanks are still capturing genetic signal.

## Conclusions

This study identified 5 genome-wide significant loci for ET in a meta-analysis of 7177 individuals with ET and 475 877 control individuals, suggesting that approximately 18% of ET heritability might be explained by common variation. The meta-analysis implicated genes such as *BACE2* and reinforced the importance of the cerebellum for the etiology of ET. The results also point toward approximately 30% shared common variant overlap with PD, and no genetic evidence for ET as a risk factor for PD.

## Notes

Supplement 1. eMethods eFigure 1. QQ plot of GWAS meta-analysis eFigure 2. QQ plot of ET gene-level association study eFigure 3. Manhattan plot of ET gene-level association study eFigure 4. Gene enrichment across GTEx 53 samples eFigure 5. Gene enrichment across BrainSpan cohort eTable 1. Genetic correlation between cohorts with Neff greater than 5000 eTable 2. Heterogeneity of the lead SNPs for ET GWAS eTable 7. Significant partitioned heritability by functional annotation eTable 8. Gene-level pathway enrichment hits for ET eReferences

Supplement 2. eTable 3. Significant transcriptome-wide association hits for ET

Supplement 3. eTable 4. Significant brain omnibus transcriptome-wide association study

Supplement 4. eTable 5. Finemapping ET transcriptome-wide association hits

Supplement 5. eTable 6. Significant eQTL mapping of top ET loci

Supplement 6. eTable 9. Genetic correlations with ET

Supplement 7. eTable 10. ET GWAS conditioned by PD

Supplement 8. eTable 11. Multi-trait analysis of ET, PD and Depression

Supplement 9. eTable 12. Summary statistics
