# Supplementary material for: Association of Essential Tremor With Novel Risk Loci: A Genome-Wide Association Study and Meta-analysis
Source: JAMA Neurol. 2022 Jan 4;79(2):1–9. doi: 10.1001/jamaneurol.2021.4781 (PMC8728658; doi:10.1001/jamaneurol.2021.4781)
Supplement: Supplement 1. — eMethods eFigure 1. QQ plot of GWAS meta-analysis eFigure 2. QQ plot of ET gene-level association study eFigure 3. Manhattan plot of ET gene-level association study eFigure 4. Gene enrichment across GTEx 53 samples eFigure 5. Gene enrichment across BrainSpan cohort eTable 1. Genetic correlation between cohorts with Neff greater than 5000 eTable 2. Heterogeneity of the lead SNPs for ET GWAS eTable 7. Significant partitioned heritability by functional annotation eTable 8. Gene-level pathway enrichment hits for ET eReferences [file jamaneurol-e214781-s001.pdf]

# Supplemental Online Content

Liao C, Castonguay C-E, Heilbron K, et al; 23andMe Research Team. Association of essential tremor with novel risk loci: a genome-wide association study and meta-analysis. *JAMA Neurol*. Published online January 4, 2022. doi:10.1001/jamaneurol.2021.4781

**eMethods.**

**eFigure 1.** QQ plot of GWAS meta-analysis

**eFigure 2.** QQ plot of ET gene-level association study

**eFigure 3.** Manhattan plot of ET gene-level association study

**eFigure 4.** Gene enrichment across GTEx 53 samples

**eFigure 5.** Gene enrichment across BrainSpan cohort

**eTable 1.** Genetic correlation between cohorts with Neff greater than 5000

**eTable 2.** Heterogeneity of the lead SNPs for ET GWAS

**eTable 7.** Significant partitioned heritability by functional annotation

**eTable 8.** Gene-level pathway enrichment hits for ET

**eReferences.**

This supplemental material has been provided by the authors to give readers additional information about their work.

© 2022 Liao et al. *JAMA Neurology.*

**eMethods.**

**Detailed description of clinical samples**

The two clinical cohort as described in the methods are partitioned by genotyping array. The first clinical cohort was ascertained and genotyped on the Axiom Genome-wide CEU 1 Array by Affymetrix as described in Müller et al. (2016)[1]. Briefly, samples from Kiel, Tuebingen, Innsbruck, Baylor, Columbia/Yale, Montreal, VCU, NIH, Mayo Clinic Florida, and the University of Saskatchewan were part of clinical cohort one (genotyped on Axiom array). The second clinical cohort was ascertained and genotyped on the Illumina GSA array. Samples were from Kiel, Montreal, Mayo Clinic, University of Saskatchewan, University of Navarra, CARTaGENE, Hopsital Universitario del Sureste (Arganda del Rey) / University of Extremadura, and University of Genoa. Both clinical cohorts are characterized by the genotyping array (clinical cohort 1 – Axiom Array, clinical cohort 2 – Illumina Array). Briefly, the contributing institutions diagnoses' have been summarized below.

**Kiel**

Patients were recruited within the "Population Based Assessment of Genetic Risk Factors for Essential Tremor (PopGen ET)" cross-sectional study from the Department of Neurology of Kiel University hospital and from newspaper calls for patients with tremor disorders from 2001 to 2013. Participants were all of German origin. Patients were diagnosed with essential tremor (ET) according to consensus criteria from the Movement Disorder Society (MDS) by one of five movement disorder experts. Only patients with definite or probable ET were genotyped in order to increase diagnostic certainty. Patients with a history of other neurological symptoms were excluded, including symptoms of Parkinson's disease. Informed consent was obtained for all recruited patients and study approval was given by the local ethics committee.

**Tuebingen**

Patients were recruited by referral to either a specialised outpatient clinic or the neurological ward for movement disorders of the department of neurodegeneration in Tuebingen. Blood samples were collected after obtaining signed informed consent. Familial history of tremor prompted examination and collection of samples from other family members. Only 'definite' or 'probable' cases of ET were retained based on criteria from the Tremor Research Investigational Group

© 2022 Liao et al. *JAMA Neurology.*

(TRIG), whilst excluding secondary causes of tremor. Sample collection was approved by the local ethics committee of Tuebingen.

**Innsbruck**

Diagnosis and recruitment of ET patients was based on the MDS Consensus Criteria for Classical Essential Tremor. Patients were recruited at the movement disorder clinical in the Department of Neurology, Medical University Innsbruck. All patients underwent clinical examination, recording of demographic and clinical information, as well as recording of treatments received, family history and data on race and ethnic group. Written informed consent of all patients was obtained. The clinical-epidemiological study received approval by the Ethics Committee. Excluded were patients with abnormal neurologic signs, enhanced physiological tremor, recent exposure to tremorogenic drugs or drug withdrawal, direct or indirect injury to the nervous system within 3 months preceding tremor onset, or clinical history of psychogenic tremor, sudden onset or stepwise deterioration of tremor.

**Baylor College of Medicine (BCM)**

TRIG criteria for 'definite' or 'probable' ET were used for diagnosis of referred patients to the movement disorders clinic. Exclusion criteria were abnormal neurologic signs, enhanced physiological tremor, recent exposure to tremorogenic drugs or drug withdrawal states, direct or indirect trauma to the nervous system within 3 months preceding tremor onset, historic or clinical evidence of psychogenic tremor and evidence of sudden onset or stepwise deterioration of tremor.

**Columbia/Yale**

Patients were recruited at the Neurological Institute of New York, Columbia University as part of a clinical-epidemiological study. The study was approved by the Institutional Review Board at Columbia University. Signed informed consent was obtained from all participants.

Patient assessment included a demographic and medical history questionnaire, information on first- and second-degree relatives with nonspecific tremor, ET or Parkinson's disease, self-reported tremor onset age, videotaped neurological examination, as well as self-reported data on race and ethnicity. Diagnosis was reconfirmed following the viewing of the videotaped neurological examination by a senior neurologist specializing in movement disorders (E.D.L). Exclusionary

© 2022 Liao et al. *JAMA Neurology.*

criteria for ET included bradykinesia or other signs of parkinsonism, except isolated rest tremor. No patients or controls had signs of amyotrophic lateral sclerosis (ALS) or a history of ALS.

**Montreal**

Ethical approval for the recruitment of ET patients was obtained from multiple institutes: Centre de recherche du Centre hospitalier de l'Université de Montréal (CIRHUM; project no: ND043076), Centre hospitalier affilié universitaire de Québec (CHA; project no: PEJ-280) and the Sainte Justine University Hospital Center (CHSJ; project no: 2352). Blood samples used for DNA extraction and cell line establishment were collected from ET patients after obtaining written consent. ET diagnoses were reviewed by a senior neurologist. Exclusion criteria were exaggerated physiological tremor, other neurological deficits (parkinsonism, polyneuritis, others), and presence of orthostatic or psychogenic-like tremors.

**Virginia Commonwealth University (VCU)**

ET patients were given a research diagnosis based on published research criteria following examination by a movement disorder neurologist or review of longitudinal movement disorder clinical notes with examination, medical response data, handwriting and research interview data. All patients included in the study gave their written consent. Self-identified ethnicity was also obtained from all participants. No known related samples between or within groups were present in the study. Patients with diagnoses of both ET and Parkinson's disease were excluded, as well as patients with signs of dystonia or other significant neurological diagnoses (history of or evidence upon examination). Parkinson's diagnoses were based on UK Brain Bank criteria. Control subjects were recruited based on availability of clinical research control samples and fulfilment of previously mentioned criteria (see above). Exclusion of control subjects were based on reported personal or family history of tremor, ET, Parkinson's, dystonia and other significant neurological diagnoses.

**National Institute of Health (NIH)**

ET patients that met the MDS Consensus Criteria for Classical Essential Tremor were recruited within the National Institutes of Neurological Disorders and Stroke, Intramural Research Program

© 2022 Liao et al. *JAMA Neurology.*

under a genotype-phenotype protocol. This protocol was approved by the NIH Combined Neurosciences IRB. Written consent was obtained for all participant prior to recruitment.

**Mayo Clinic Florida**

Patients and control subjects were recruited under the Mayo Clinic IRB Committee approved protocol, IRB#: 1087-98, P.I. Zbigniew K. Wszolek, M.D. entitled: "Clinical and Genetic Studies of Neurodegenerative Syndromes, Dystonia, and Restless Leg Syndrome". All patients and controls signed the consent form. Material Transfer Agreement specifying the legal conditions of collaboration between Mayo Clinic Florida (MCF) and McGill University was developed prior to transfer of de-identified data and DNA samples from MCF to McGill University. The patients were diagnosed with essential tremor (ET) by movement disorders specialists according to the Movement Disorders Society ET diagnostic criteria. Both sporadic and familial cases were collected. The patients with other than ET neuroglial symptoms and signs were excluded from this study. Also the patients in which ET was part of a broader phenotype and the patients who first presented with ET and later develop additional clinical/neurological features such as rest tremor, rigidity, bradykinesia, dystonia, tremor, chorea, and others were excluded from this study. The controls were mainly opportunistic (not specifically sought for this study), and mainly included spouses, other family members, and care-givers. A subset of controls was recruited through electrodiagnostic laboratory and included the patients who were electro-diagnostically studied for the median neuropathy at the wrist, ulnar neuropathy at the elbow, peroneal neuropathy at the fibular head, or minor complains consistent with peripheral neuropathy.

**University of British Columbia (UBC) / University of Saskatchewan**

Patients were recruited from the Movement Disorder Clinic Saskatchewan (MDCS). Diagnosis was made based on postural and/or kinetic tremor of upper limbs or head tremor with no other known neurological cause. In certain cases, brain pathological studies were performed following autopsy to identify known tremor causes.

**University of Navarra (Spain)**

© 2022 Liao et al. *JAMA Neurology.*

The study was approved by the University of Navarra's Ethical Committee and all informed consent was obtained to patients prior to their inclusion in the study. Patients were diagnosed by a movement disorder neurologist with either 'definite' or 'probable' ET based on current criteria used by the Department of Neurology from the Clinica Universidad de Navarra, Pamplona, Spain. Only one affected subject from each family with the familial form of the disease were included in the study. Controls were unrelated healthy subjects or spouses from PD cases, recruited within the same university center or from the community. Exclusion criteria were patients with other neurological disorders or a positive family of neurodegenerative disease.

**Hopsital Universitario del Sureste (Arganda del Rey) and University of Extremadura**

Participants were recruited from the University Hospitals of Sureste (Arganda del Rey, Madrid) and *Principe de Asturias* (Alcalá de Henares, Madrid). Diagnosis of ET patients were based on 'definite' ET criteria from MDS. Only patients without other neurological disorders and without thyroid dysfunction were included. All ET patients had at least 1 first-degree relative with ET and only 1 family member per family was included. Control particpiants were recruited from the Infanta Cristina University Hospital (Badajoz) and from the Clinica Universitaria de Navarra (Pamplona). These were healthy, gender-matched, unrelated participants without tremor. Informed consent was obtained before inclusion in the study. Ethical approval was given by the Ethics Committees of the Infanta Cristina University Hospital and the *Principe de Asturias* University Hospital.

**University of Genoa (Genova, Italy)**

ET patients with 'definite' or 'probable' ET based on criteria from the Tremor Investigation Group[2,3] were recruited at the collaborating Neurological Centres (Naples and Bologna) by specialized neurologists. Assessment of patients involved neurological examination, identification of tremor type and its topography as well as recording of associated signs or symptoms, drugs in use, videotaping and other paraclinical investigations. Self-reported age of onset was recorded. Samples from patients and relatives (when possible) were obtained after written informed consent was given. Age- and gender-matched controls were recruited among the spouses of Italian ET patients or other subjects without neurological dysfunctions (as assessed by the same neurologists and protocols previously mentioned).

© 2022 Liao et al. *JAMA Neurology.*

**CARTaGENE**

The CARTaGENE cohort was used as additional controls for the study. CARTaGENE is population-based biobank of individuals from Quebec, Canada[4]. It is a long-term cohort of over 20,000 participants that consent to visiting assessment sites to provide health and sociodemographic information. Individuals without neurological or psychiatric disorders were used as controls. Further details about CARTaGENE are described in Awadalla et al (2013)[4].

**Quality control and association analyses for clinical cohorts**

The samples from both clinical cohorts 1 and 2 that were genotyped on the Axiom CEU Array and Illumina GSA array respectively. and followed standardized quality control (QC), imputation, and post-imputation QC. Briefly, samples were removed if >2% missingness, autosomal heterozygous deviation (Fhet <0.2), and failed sex check[5]. Low quality SNPs were removed based on Hardy-Weinberg Equilibrium (P>1E-6), SNP missingness < 0.02 after sample removal. Samples were mapped against the 1000 Genomes Project phase 3 reference panel after pruning and removing SNPs from high-LD regions, and only individuals of inferred- European ancestry were retained[6]. No relatedness filter was done because a linear-mixed model was used subsequently to account for relatedness. Imputation was done using the Sanger Imputation Server with Eagle v2.3.5 and the Haplotype Reference Consortium Reference Panel v1.1[7,8]. A Bayesian linear-mixed model was done using BOLT-LMM 2.3.4 including 20 principal components (PCs) and sex as covariates to accelerate convergence[9]. The non-infinitesimal model was used if there was an increase in power.

**Description of population-level cohorts**

Two separate biobanks, 23andMe and the UK BioBank were used for the study.

**23andMe**

The 23andMe samples consisted of individuals who sent saliva samples to 23andMe Inc. and agreed to partake in research and answered questions related to ET. Participants involved provided informed consent and answered questions in accordance to their human subjects' protocol, which was approved and reviewed by Ethical and Independent Review services, an AAHRPP-accredited institutional review board. 23andMe provided summary statistics of their ET GWAS that included

© 2022 Liao et al. *JAMA Neurology.*

cases that responded “yes” to the question “Have you ever been diagnosed with a neurological condition?” and later indicated that they had been diagnosed with ET or indicated that they had been diagnosed with Parkinson’s disease but were later re-diagnosed with ET. Cases were excluding individuals genotyped on the 23andMe v1 genotyping chip and individuals younger than 30 years old.

Controls were recruited from all eligible participants. The age, sex, genotyping platform and survey response time were matched against the cases that met the same criteria but excluded cases that were genotyped on the 23andMe v1 and subjects under 30 years old. Controls were iteratively matched by age, gender and genotyping platform. This was done by splitting individuals into 20 age bins, each containing an approximately equal number of ET cases, date of entry to the 23andMe research cohort (by splitting individuals into 20 bins, each containing an approximately equal number of individuals), genotyping platform, and sex.

Participants were restricted to a set of individuals with European ancestry through an analysis of local ancestry. Briefly, the algorithm partitions phased genomic data into intervals of 300 SNPs. Iteratively for each window, a support vector machine (SVM) was used to characterize each haplotype into reference populations (1000 Genomes, HapMap, Human Genome Diversity Project, 23andMe customers reporting four grandparents from same country). To account for relatedness, a segmental identity-by-descent (IBD) estimation algorithm was used. Individuals that shared roughly 20% of the genome were considered related. When selecting for case/control, cases were preferentially retained. Imputation was done with an imputation panel combining the 1000 Genomes Phase 3 haplotypes with the UK10K reference panel[10]. Participants from the v5 platform and prior, the tool Finch was used to phase participant data. For samples from the v5 array, Eagle2 was used.

Association tests for genotyped data was done using a logistic regression assuming additive effects. For imputed data, the dosage was used over hard-call genotypes. Quality control was done independently for dosage and imputed data. The directly genotyped SNPs were flagged for failed QC if they were only genotyped on v1 or v2 platforms due to smaller sample size. SNPs with a Hardy-Weinberg (P<10E-20) or a call rate of <90% were flagged. Any SNPs that were flagged by ANOVA of genotypes against a factor dividing genotyping date into 20 roughly equal bins were

© 2022 Liao et al. *JAMA Neurology.*

additionally flagged. SNPs with large sex-effects were additionally flagged (ANOVA of genotypes, r2 >0.1). Finally, SNPs with probes that match multiple genomic positions in the hg19 reference genome were flagged. For the imputed data, any SNPs with an rsq < 0.30 or evidence of batch effect were flagged based on an ANOVA F-test of SNP dosage against a factor of v4 and v5 platform (P<10E-50). Across all data, SNPs that do not have a sample size >20% of the total GWAS were flagged. Furthermore, SNPs that did not converge during logistic regression.

**UK BioBank**

Cases were individuals that answered "yes" to have benign / essential tremor in the UK Biobank. Controls were individuals that answered "no" to the question. A total of 216 cases and 395,209 controls were included. Summary statistics were ascertained from Zhou et al. (2018) (https://www.leelabsg.org/resources), which were publicly available summary statistics of UKBiobank phecode binary phenotypes that were ran using SAIGE (Scalable and Accurate Implementation of Generalized mixed model). SAIGE was used due to the large case-control imbalance. Data analysis can be found in detail in Zhou et al. (2018)[11].

© 2022 Liao et al. *JAMA Neurology.*

**Supplementary Figures**

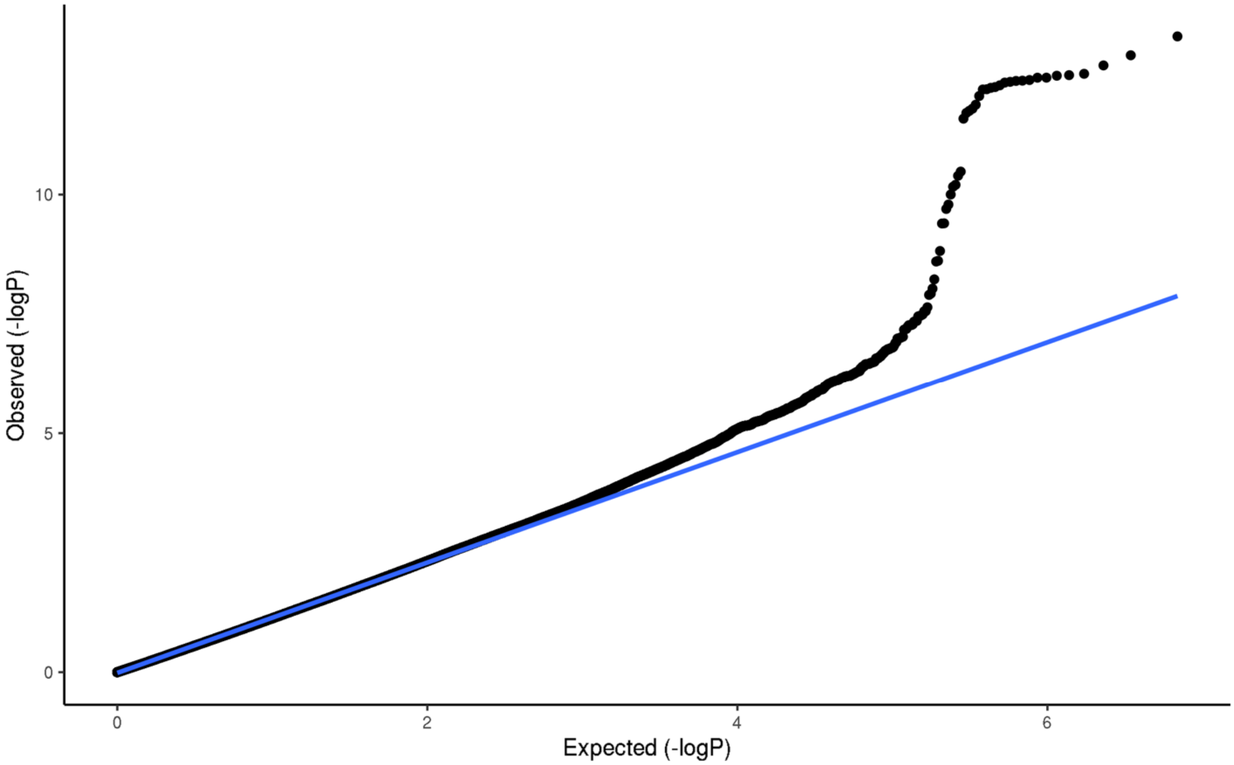


**Supplementary Figure 1. QQ plot of GWAS meta-analysis.**

© 2022 Liao et al. *JAMA Neurology.*

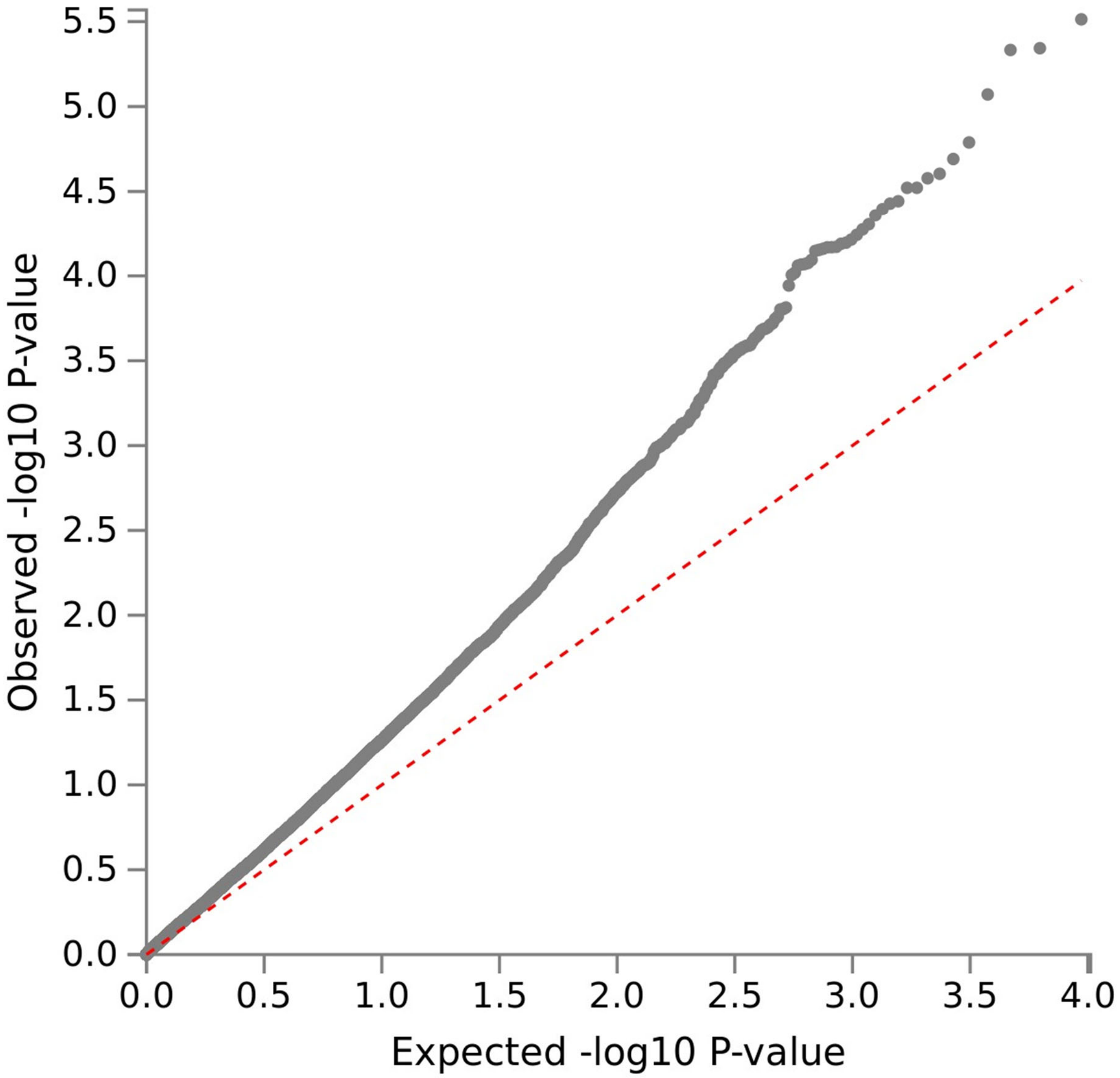


**Supplementary Figure 2. QQ plot of ET gene-level association study.**

© 2022 Liao et al. *JAMA Neurology.*

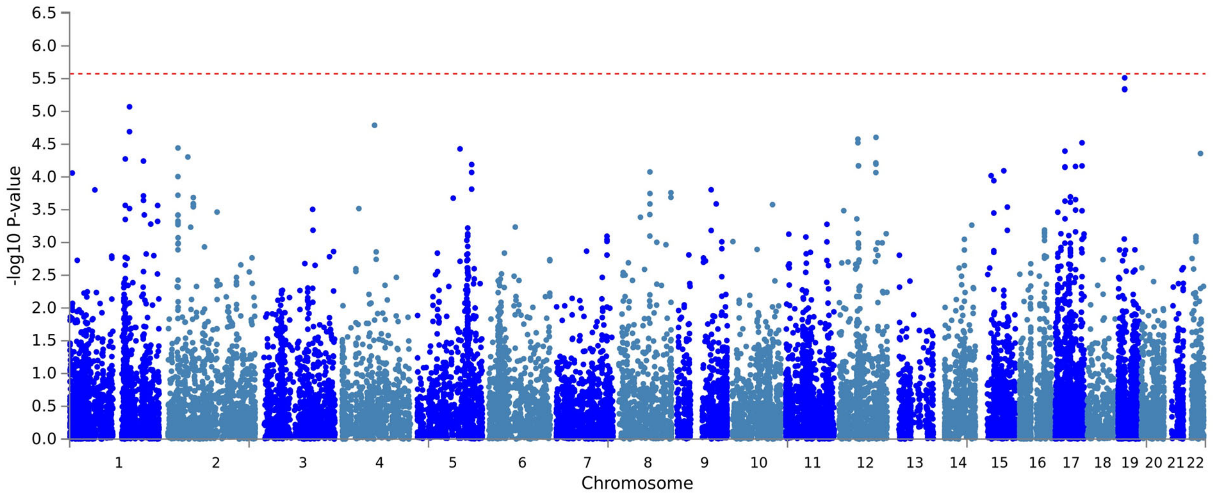


**Supplementary Figure 3. Manhattan plot of ET gene-level association study.**

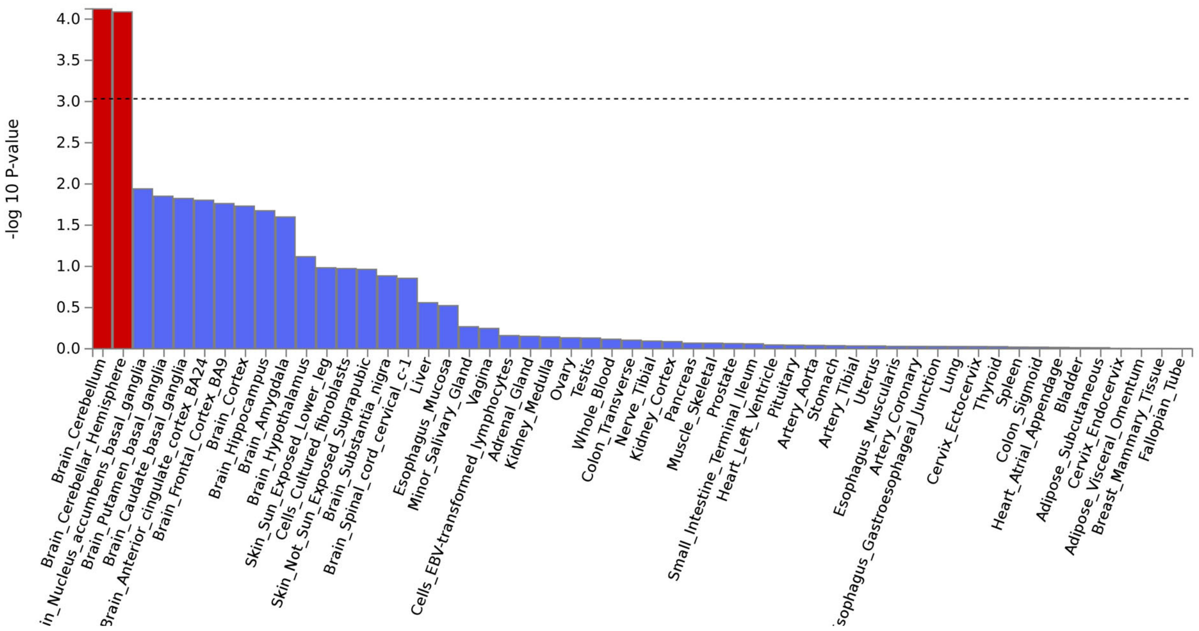


**Supplementary Figure 4. Gene enrichment across GTEx 53 samples.**

© 2022 Liao et al. *JAMA Neurology.*

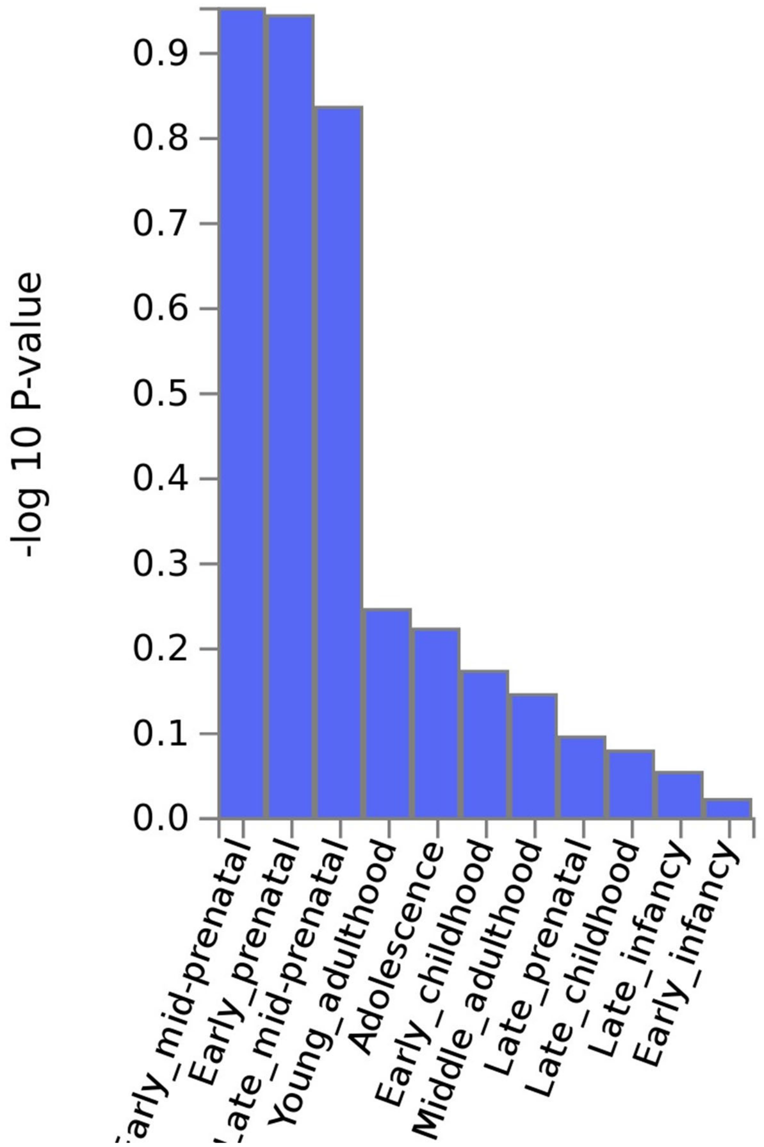


**Supplementary Figure 5. Gene enrichment across BrainSpan cohort.**

© 2022 Liao et al. *JAMA Neurology.*

**Supplementary Table 1. Genetic correlation between cohorts with Neff greater than 5000**

| Cohort 1 | Cohort 2 | rg | se | z | p |
|---|---|---|---|---|---|
| ET 2016 | 23andMe | 0.9584 | 0.148 | 6.4772 | 9.34E-11 |
| ET 2020 | 23andMe | 0.5235 | 0.1362 | 3.8435 | 1.21E-04 |
| ET 2020 | ET 2016 | 0.8761 | 0.2047 | 4.2804 | 1.87E-05 |

**Supplementary Table 2. Heterogeneity of the lead SNPs for ET GWAS.**

| CHR | POS (hg19) | rsID | Alleles (Eff/Ref) | Direction | Het ChiSq | P |
|---|---|---|---|---|---|---|
| 1 | 117532790 | rs1127215 | C/T | +++- | 2.362 | 0.5007 |
| 4 | 24362541 | rs17590046 | C/T | ---- | 7.377 | 0.06079 |
| 5 | 67827456 | rs28562175 | C/T | ---- | 2.084 | 0.5552 |
| 18 | 37207175 | rs1945016 | G/T | ++++ | 6.313 | 0.09734 |
| 21 | 42520134 | rs9980363 | C/T | ++++ | 2.165 | 0.5388 |

**Supplementary Table 7. Significant partitioned heritability by functional annotation.**

| Annotation | Proportion of h2 +/- SE | Enrichment | Std Error | p | $P_{bonf}$ |
|---|---|---|---|---|---|
| H3K9ac peaks | 0.385 (0.10) | 10.02 | 2.67 | 0.0003281 | 0.0249 |
| GERP NSL2 | 2.884 (0.29) | 1.627 | 0.171 | 0.00036225 | 0.0275 |
| H3K27ac | 0.6534 (0.068) | 1.554 | 0.162 | 0.00055661 | 0.0423 |

**Supplementary Table 8. Gene-level pathway enrichment hits for ET.**

| Gene set | N genes | Beta | Beta STD | Beta SE | P | Pbon |
|---|---|---|---|---|---|---|
| Curated_gene_sets:reactome_p75ntr_regulates_axonogenesis | 9 | 1.3068 | 0.028607 | 0.28864 | 3.01E-06 | 0.04653871 |
| Curated_gene_sets:kegg_glycosphingolipid_biosynthesis_ganglio_series | 15 | 0.99468 | 0.028106 | 0.23005 | 7.72E-06 | 0.11948696 |
| Curated_gene_sets:davicioni_pax_foxo1_signature_in_arms_dn | 19 | 0.79079 | 0.025146 | 0.19813 | 3.30E-05 | 0.51076666 |
| Curated_gene_sets:delaserna_targets_of_myod_and_smarca4 | 11 | 0.81766 | 0.019787 | 0.20734 | 4.03E-05 | 0.62391526 |
| Curated_gene_sets:biocarta_trka_pathway | 14 | 0.6794 | 0.018547 | 0.17731 | 6.38E-05 | 0.98835156 |
| Curated_gene_sets:chemnitz_response_to_prostaglandin_e2_up | 137 | 0.2568 | 0.021858 | 0.067093 | 6.50E-05 | 1 |
| GO_bp:go_negative_regulation_of_fat_cell_differentiation | 44 | 0.50676 | 0.024506 | 0.13376 | 7.60E-05 | 1 |
| GO_bp:go_platelet_derived_growth_factor_receptor_signaling_pathway | 53 | 0.4015 | 0.021304 | 0.10637 | 8.04E-05 | 1 |
| GO_bp:go_thrombin_activated_receptor_signaling_pathway | 12 | 0.97077 | 0.024536 | 0.25588 | 7.45E-05 | 1 |
| GO_cc:go_axon | 574 | 0.13128 | 0.022603 | 0.035157 | 9.45E-05 | 1 |

© 2022 Liao et al. *JAMA Neurology.*

**Code**

```
#### QC ####
plink \
--bfile et \
--geno 0.02 \
--maf 0.01 \
--hwe 0.000001 \
--make-bed \
--out clean.qc.et


#### SEX CHECK ####
plink \
--bfile clean.qc.et \
--check-sex ycount 0.2 0.8 0 1 \
--out sex_check


#### ANCESTRY PREDICTIONS ####
python -m peddy --plot -p 4 --prefix mystudy clean.qc.et clean.qc.et.ped


#### PRUNE ####
plink2 --bfile clean.qc.et \
 --indep-pairwise 200 100 0.2 \
 --out pruned.clean.qc.et.temp


plink2 --bfile clean.qc.et \
--extract pruned.clean.qc.et.temp.prune.in \
--indep-pairwise 200 100 0.2 \
```

© 2022 Liao et al. *JAMA Neurology.*

```
--out pruned.clean.qc.et


#### HETEROGENEITY TEST ####
plink \
--bfile runed.clean.qc.et \
--ibc \
--out ibc.pruned.clean.qc.et


#### PCA ####
plink2 --bfile pruned.clean.qc.et \
--pca approx 20 \
--maf 0.01 \
--out pca.pruned.clean.qc.et


#### BOLT EXAMPLE ####

cd /lustre03/project/6004655/COMMUN/runs/cliao15/runs/ET_Genotyping_Cases/
/home/cliao15/software/BOLT-LMM_v2.3.4/bolt \
	--lmmForceNonInf \
	--LDscoresFile=/home/cliao15/software/BOLT-
LMM_v2.3.4/tables/LDSCORE.1000G_EUR.tab.gz \
	--qCovarCol=PC{1:20} \
	--covarCol=sex \
	--covarCol=plate \
	--covarFile=pca.sex.platecovar.6mill.ETold.ETnew.CAG_2020-06-02.txt \
	--numThreads 80 \
	--LDscoresMatchBp \
```

© 2022 Liao et al. *JAMA Neurology.*

```
    --geneticMapFile=/home/cliao15/software/BOLT-
LMM_v2.3.4/tables/genetic_map_hg38_withX.txt.gz \
    --bfile=clean.qc.et \
    --statsFile=noafr.eas.merged.allCAG.1800ET.2020-06-02_v2_6mill.bolt.assoc.stat \
    --phenoFile=affect.merged.oldET.newET.CAG.6mil_2020-06-02.txt \
    --phenoCol=affect \
    --maxMissingPerSnp 1 \
    --modelSnps=pruned.snps \
    2>>bolt.log


#### META-ANALYSIS GWAS ####

# UNCOMMENT THE NEXT LINE TO ENABLE GenomicControl CORRECTION
# GENOMICCONTROL ON

# === DESCRIBE AND PROCESS THE FIRST INPUT FILE ===
MARKER snp
ALLELE alleleA alleleB
EFFECT effect
STDERR stderr
PVALUE pvalue
DEFAULTWEIGHT 12960.45
PROCESS
/lustre03/project/6004655/COMMUN/runs/cliao15/runs/ET_Genotyping_Cases/combine.all/23and
me/hg19_sepallele.clean_23andme_ET3400_noquotes_2020-05-27.txt

# === THE SECOND INPUT FILE HAS THE SAME FORMAT AND CAN BE PROCESSED
IMMEDIATELY ===
MARKER snp_hg19
ALLELE ALLELE0 ALLELE1
```

© 2022 Liao et al. *JAMA Neurology.*

```
EFFECT transformed_beta
STDERR transformed_se
PVALUE P_BOLT_LMM_INF
DEFAULTWEIGHT 5982.26
PROCESS
/lustre03/project/6004655/COMMUN/runs/cliao15/runs/ET_Genotyping_Cases/impute.combined/hrc/commonid.pruned.merged.maf05hwe1e6.geno02.all.dose.bgen_bgensnps.bolt.assoc.stat.correctbeta

# === DESCRIBE AND PROCESS THE THIRD INPUT FILE ===
MARKER snp_hg19
ALLELE ALLELE0 ALLELE1
EFFECT transformed_beta
STDERR transformed_se
PVALUE P_BOLT_LMM
DEFAULTWEIGHT 5344.44
PROCESS
/lustre03/project/6004655/COMMUN/runs/cliao15/runs/simonGWAS_ET/Imputed2/ETimputationHap.vcfs_CLEANED/vcfs/sorted_concatenated_cleaned_hwe1e6_geno002_ETimputed_hap_20180825_info03_plinkbed_imputeMAF001hwe1e6_bolt_20180901_20pca.metawithID.correctbeta

# === DESCRIBE AND PROCESS THE FOURTH INPUT FILE ===
MARKER snp_hg19
ALLELE ref alt
EFFECT transformed_beta
STDERR transformed_se
PVALUE pval
DEFAULTWEIGHT 863.528
PROCESS
/lustre03/project/6004655/COMMUN/runs/cliao15/runs/ET_Genotyping_Cases/meta.analysis/UKBB.SAIGE.GWAS.snpid.correctbeta.tsv
```

© 2022 Liao et al. *JAMA Neurology.*

ANALYZE

OUTFILE et.meta.analysis.txt

```
#### TWAS ####
file=et.meta.analysis.txt
for tissue in `cat tissue.twas.list` ;  do
for i in {1..22};
do Rscript ~/software/fusion_twas-master/FUSION.assoc_test.R \
        --sumstats $file \
        --perm 10000 \
        --ref_ld_chr ~/software/fusion_twas-master/LDREF/1000G.EUR. \
        --weights ~/software/fusion_twas-master/weights/$tissue \
        --weights_dir ~/software/fusion_twas-master/weights \
        --chr $i \
        --out fusion_et/fusion.${tissue}.${file}.chr${i} ; done ; done
```

```
#### MTAG ####
python mtag/mtag.py \
--sumstats neff.et.meta.analysis.txt,depression.gwas.txt,pd.gwas.txt \
--out mtag.gwas \
--n_min 0.0 \
--fdr \
--stream_stdout &
```

```
#### mtCOJO ####
--bfile /home/cliao15/data/1000G_European_withRSID_GRCh37/merged_1000G.Eur.QC.rsID
--mtcojo-file mtcojo_summary_data.txt
--ref-ld-chr /home/cliao15/runs/data/eur_w_ld_chr/
```

© 2022 Liao et al. *JAMA Neurology.*

```
--w-ld-chr /home/cliao15/runs/data/eur_w_ld_chr/
--out ETcondPD

#### LDSC ####
munge_sumstats.py \
--sumstats neff.et.meta.analysis.txt \
--out et.meta.analysis \
--merge-alleles w_hm3.snplist

ldsc.py \
--h2 et.meta.analysis.sumstats.gz \
--ref-ld-chr eur_w_ld_chr/ \
--w-ld-chr eur_w_ld_chr/ \
--out et_h2
```

**References for Supplementary Note**


1. Müller, S. H. *et al.* Genome-wide association study in essential tremor identifies three new loci. *Brain* **139**, 3163–3169 (2016).
2. Deuschl, G. *et al.* Consensus statement of the Movement Disorder Society on tremor. in *Movement Disorders* (1998). doi:10.1002/mds.870131303.
3. Bhatia, K. P. *et al.* Consensus Statement on the classification of tremors. from the task force on tremor of the International Parkinson and Movement Disorder Society. *Mov. Disord.* (2018) doi:10.1002/mds.27121.
4. Awadalla, P. *et al.* Cohort profile of the CARTaGENE study: Quebec's population-based biobank for public health and personalized genomics. *Int. J. Epidemiol.* **42**, 1285–99 (2013).
5. Anderson, C. A. *et al.* Data quality control in genetic case-control association studies. *Nat. Protoc.* **5**, 1564–1573 (2010).
6. Price, A. L. *et al.* Long-Range LD Can Confound Genome Scans in Admixed Populations. *American Journal of Human Genetics* (2008) doi:10.1016/j.ajhg.2008.06.005.
7. Loh, P. R., Palamara, P. F. & Price, A. L. Fast and accurate long-range phasing in a UK Biobank cohort. *Nat. Genet.* (2016) doi:10.1038/ng.3571.


© 2022 Liao et al. *JAMA Neurology.*

8. McCarthy, S. *et al.* A reference panel of 64,976 haplotypes for genotype imputation. *Nat. Genet.* **48**, 1279–1283 (2016).
9. Loh, P.-R. *et al.* Efficient Bayesian mixed-model analysis increases association power in large cohorts. *Nat. Genet.* **47**, 284–290 (2015).
10. Huang, J. *et al.* Improved imputation of low-frequency and rare variants using the UK10K haplotype reference panel. *Nat. Commun.* **6**, 8111 (2015).
11. Zhou, W. *et al.* Efficiently controlling for case-control imbalance and sample relatedness in large-scale genetic association studies. *Nat. Genet.* (2018) doi:10.1038/s41588-018-0184-y.

© 2022 Liao et al. *JAMA Neurology.*
